# Proposed Diffusion Mechanism and Microstructural Evolution in MIG Braze-Welded Cu–Austenitic Steel Joints

**DOI:** 10.3390/ma19214685

**Published:** 2026-11-03

**Authors:** Bożena Szczucka-Lasota, Klaudiusz Gołombek, Tomasz Węgrzyn, Oktawian Bialas, Bogusław Łazarz, Patrycja Lasota

**Affiliations:** 1 Faculty of Transport and Aviation Engineering, Silesian University of Technology, Zygmunta Krasińskiego Str. 8, 40-019 Katowice, Poland; boguslaw.lazarz@polsl.pl; 2 Faculty of Mechanical Engineering, Silesian University of Technology, Konarskiego 18A, 44-100 Gliwice, Poland; klaudiusz.golombek@polsl.pl (K.G.); oktawian.bialas@polsl.pl (O.B.); 3 Faculty of Biomedical Engineering, Silesian University of Technology, Roosvelta 40, 41-800 Zabrze, Poland; pl315065@student.polsl.pl

**Keywords:** MIG braze welding, Cu–steel dissimilar joint, AISI 304 stainless steel, CuSi3 filler metal, interfacial diffusion, EBSD

## Abstract

**Highlights:**

- Cu–steel braze-welded joints with continuous interfaces were produced for potential marine applications.
- Process parameters affected wetting, interfacial continuity, and microstructural evolution of the Cu–steel joints.
- Diffusion and microstructural evolution differed at the Cu-side and steel-side interfaces.
- The joint exhibited an asymmetric interfacial structure resulting from the different properties and interactions of the two base materials.
- A formation mechanism linking wetting, diffusion, and asymmetric interfacial microstructure was proposed based on experimental observations.

**Abstract:**

Dissimilar joints between copper and austenitic stainless steel are relevant to marine and shipbuilding systems in which corrosion resistance, thermal conductivity, and structural integrity are required. This study investigated the formation, microstructure, and mechanical performance of Cu–austenitic steel joints produced by MIG braze welding, with particular emphasis on wetting, interfacial diffusion, and the asymmetric response of the two base materials. The joints were produced within a process window of 150–170 A, 19–21 V, and 220–320 mm/min. Their quality and structure were evaluated using visual testing (VT), penetrant testing (PT), tensile testing, light microscopy (LM), scanning electron microscopy with energy-dispersive X-ray spectroscopy (SEM/EDS), and electron backscatter diffraction (EBSD). The applied process parameters affected filler-metal spreading, joint continuity, interfacial morphology, and the occurrence of welding imperfections. Among the defect-free joints selected for tensile testing, the ultimate tensile strength ranged from 123 to 131 MPa. The highest UTS of 131 MPa was obtained for the joint produced at 170 A, 19 V, and 320 mm/min. Microscopic and compositional analyses showed that joint formation proceeded differently at the Cu-side and steel-side interfaces, reflecting differences in the thermal and metallurgical behavior of the base materials and resulting in an asymmetric interfacial structure. Based on the LM, SEM/EDS, and EBSD observations, an integrated joint-formation mechanism combining wetting, diffusion, solidification, and side-dependent microstructural evolution was proposed. The results demonstrate that MIG braze welding can produce continuous Cu–austenitic steel joints within a defined process window and that joint quality and mechanical performance depend on the specific combination of current, voltage, and travel speed.

## 1. Introduction

Braze welding of copper to austenitic steel remains a significant technological challenge due to pronounced differences in the physical and chemical properties of the materials involved. Such joints are, for example, used in the construction of shipboard systems.

Modern shipboard systems (e.g., refrigeration equipment) require joining technologies that ensure high reliability, long service life, and resistance to harsh operating conditions [1]. Shipbuilding projects increasingly utilize assemblies in which copper—used for its high thermal and electrical conductivity—must be joined with austenitic steel, valued for its corrosion resistance, mechanical strength, and stability at elevated temperatures. Typical examples include electrical systems for refrigeration equipment, pipes, and heat exchangers. Direct fusion welding of these materials remains problematic due to substantial differences in melting temperature, coefficients of thermal expansion, and thermal conductivity [2]. These differences may promote the development of residual stresses, cracking, and the formation of brittle intermetallic phases. In response to these limitations, arc-based braze welding (commonly referred to as MIG brazing when a consumable electrode wire is used) has been investigated as an alternative joining process. In this process, the filler metal is melted by the arc and transferred to the joint, whereas melting of the base materials is not an intended part of the process [3,4,5]. This distinguishes braze welding from conventional brazing, in which the filler metal is melted by an external heat source without the use of an arc, and from fusion welding, in which the base materials are intentionally melted to form the joint.

Differences in melting temperature, thermal conductivity, and the tendency to form brittle intermetallic phases mean that conventional welding often leads to reduced joint quality or premature failure. Statistical methods can be applied to evaluate joint quality and assess the influence of processing parameters on the resulting joint characteristics [6]. Under such conditions, braze welding, when carried out with appropriately selected copper-based filler metals, enables the formation of durable joints while limiting heat input and minimizing melting of the base materials [7,8].

The principal advantage of arc-based braze welding for dissimilar copper–austenitic steel joints is therefore the possibility of depositing a copper-based filler metal while limiting heat input and avoiding intentional melting of the base materials [7,8]. Nevertheless, the thermal cycle generated by the arc may cause local heating, diffusion, dissolution, or, depending on the process conditions, limited local melting at the interface. Whether such local melting occurs under the conditions investigated in the present study is therefore treated as an experimental question and is evaluated using the observed microstructural and compositional characteristics of the joint.

A key aspect of the process is the proper selection of the electrode wire, which must ensure adequate wetting of the austenitic steel, compatibility with copper, and suitable mechanical and corrosion properties of the joint [9,10]. The paper discusses commonly used filler metals for braze welding of copper–austenitic steel joints, with particular emphasis on three copper-based electrode wires in which alloying elements such as tin and silicon play a significant role. Based on this analysis, key technological recommendations affecting the quality and reliability of the joints were identified.

Braze welding, understood as a capillary joining process employing a filler metal with a melting temperature lower than that of the base materials, enables the formation of continuous and leak-tight joints. The capillary effect arises from the balance of forces [9,10]:

The wetting and spreading of the molten filler metal are important for joint formation and are influenced by the interaction between the liquid filler and the surfaces of the base materials, the viscosity and surface tension of the filler, the joint geometry, and the thermal conditions during deposition [9,10]. These phenomena can be described in terms of the following:

- Adhesion, i.e., the interaction between the liquid filler metal and the surfaces of the base materials;
- Cohesion, i.e., the internal bonding forces within the molten filler metal;
- Surface tension, where a lower wetting angle and a narrower joint gap promote improved filler metal penetration.

In contrast to conventional capillary brazing, however, the filler-metal flow in arc-based braze welding is not governed by capillary action alone. The arc, droplet transfer, gravity, surface tension, wetting, and the thermal gradient all contribute to the deposition and spreading of the molten filler metal. Consequently, the term “braze welding” is used throughout this manuscript to describe the investigated arc-based process, whereas “brazing” is reserved for processes in which the filler is melted without intentionally melting the base materials and without arc deposition.

Braze welding of copper to austenitic steel using a copper-based electrode wire alloyed with silicon relies on controlled wetting of the surfaces and uniform spreading of the filler metal, which is essential for obtaining a continuous and tight bimetallic joint. The addition of silicon (approximately 1–3%) reduces the melting temperature of the filler metal (to about 1010–1030 °C) and enhances its wettability with respect to both copper and austenitic steel [11,12,13].

The flow of the filler metal is governed by surface tension, the wetting angle, and the temperature gradient within the braze-weld. In contact with both copper and austenitic steel, a silicon-alloyed copper filler forms a continuous copper-rich phase with dispersed intermetallic precipitates [11,12,13]. Appropriate selection of process parameters—including current intensity, electrode travel speed, and joint geometry—may affect the thermal cycle, filler-metal transfer, wetting behavior, and consequently the formation of defects and the final microstructure of the joint [12,13].

A critical aspect of the mechanism governing the properties of the solder joint is the diffusion kinetics of elements at the interface [14,15,16]. Previous studies on the mechanisms governing solder joint formation have largely adopted a reductionist perspective, focusing on isolated aspects of the process and often implying the existence of a universal final mechanism. In particular, the role of the base materials in determining the course of joint formation remains insufficiently understood and is rarely examined in a systematic manner.

Based on the available scientific literature, these mechanisms can be classified into five principal categories [14,15,16,17,18,19,20,21]:

- Liquid-state diffusion (soldering/brazing);
- Solid-state diffusion (diffusion bonding);
- Intermetallic phase formation (IMC-driven);
- Dissolution-controlled mechanisms;
- IMC-free mechanisms (solid-solution or interlayer-controlled).

The literature in this area reveals a clear fragmentation of research approaches, with studies typically focusing on a single, narrowly defined aspect of the mechanism.

In Table 1, the bonding mechanisms reported in the literature are compared along with their principal limitations. The analysis presented in Table 1 indicates that existing studies on stainless steel–copper joints remain fragmented, most often addressing diffusion phenomena, intermetallic formation, or solidification processes in isolation. Moreover, the role of the base materials is frequently simplified, with the intrinsic asymmetry between the copper and austenitic steel interfaces receiving comparatively limited attention. None of the proposed mechanisms presented in Table 1 adequately account for or explain the impact of this intrinsic asymmetry arising from the dissimilar nature of the joined materials.

These observations indicate a need to examine whether the dissimilar nature of the two base materials produces noticeable differences in formation of structure on the copper and steel sides of the joint, in particular in internes between braze joint and base material. In particular, the available literature does not provide a sufficiently consistent basis for determining how the process parameters and the material asymmetry interact to influence defect formation and the resulting interfacial structure.

In response to this gap, the present work introduces an integrated approach encompassing both the models and mechanisms of solder joint formation, extended to include an explicit analysis of diffusion processes with consideration of the base materials’ influence.

Unlike previous studies, this work proposes a comprehensive framework that explicitly accounts for diffusion pathways, phase transformations, and material-dependent mechanisms on both sides of the joint, offering a more complete description of joint formation.

In the existing literature, solder joint formation mechanisms are typically presented in a simplified and fragmented manner, often assuming the dominance of a single final mechanism. As shown in the comparison in Table 1, particular attention to the influence of base materials on the progression of joint formation is limited, with analyses usually conducted asymmetrically or in a generalized way. Furthermore, prior studies generally focus on selected aspects of the phenomenon—such as diffusion pathways (mainly considered chemically) or the effect of temperature on solidification and crystallization processes (considered structurally).

Accordingly, the present study addresses three specific questions. First, how do the principal braze-welding parameters—particularly current, voltage, and electrode travel speed—affect the occurrence of braze-welding defects in copper–austenitic steel joints? Second, how does the interfacial microstructure and chemical composition differ in the interface between the base materials and metal deposit? Third, what experimental evidence from microstructural observations, and elemental distributions, supports the proposed sequence of dissolution, diffusion, solidification, and phase-formation processes during joint formation?

The objective of this study is to establish an experimentally supported description of the formation mechanisms of arc-braze-welded copper–austenitic steel joints, with particular emphasis on the effect of the asymmetric response of the two base materials. The study evaluates the following:

- The influence of current, voltage, and travel speed on joint defects;
- The microstructural and chemical differences between the copper-side and steel-side interfaces.

The proposed diffusion-related sequence is presented as an interpretation supported by microstructural and compositional observations rather than as a quantitatively validated diffusion model. The study further examines the distribution and evolution of the principal phases and alloying elements, as well as the experimental evidence for diffusion, dissolution, solidification, and phase-formation mechanisms. These results are used to assess whether local melting of either base material occurred under the investigated conditions and to evaluate the proposed sequence of joint-formation phenomena.

## 2. Materials and Production Process of Samples

**Figure 1:**
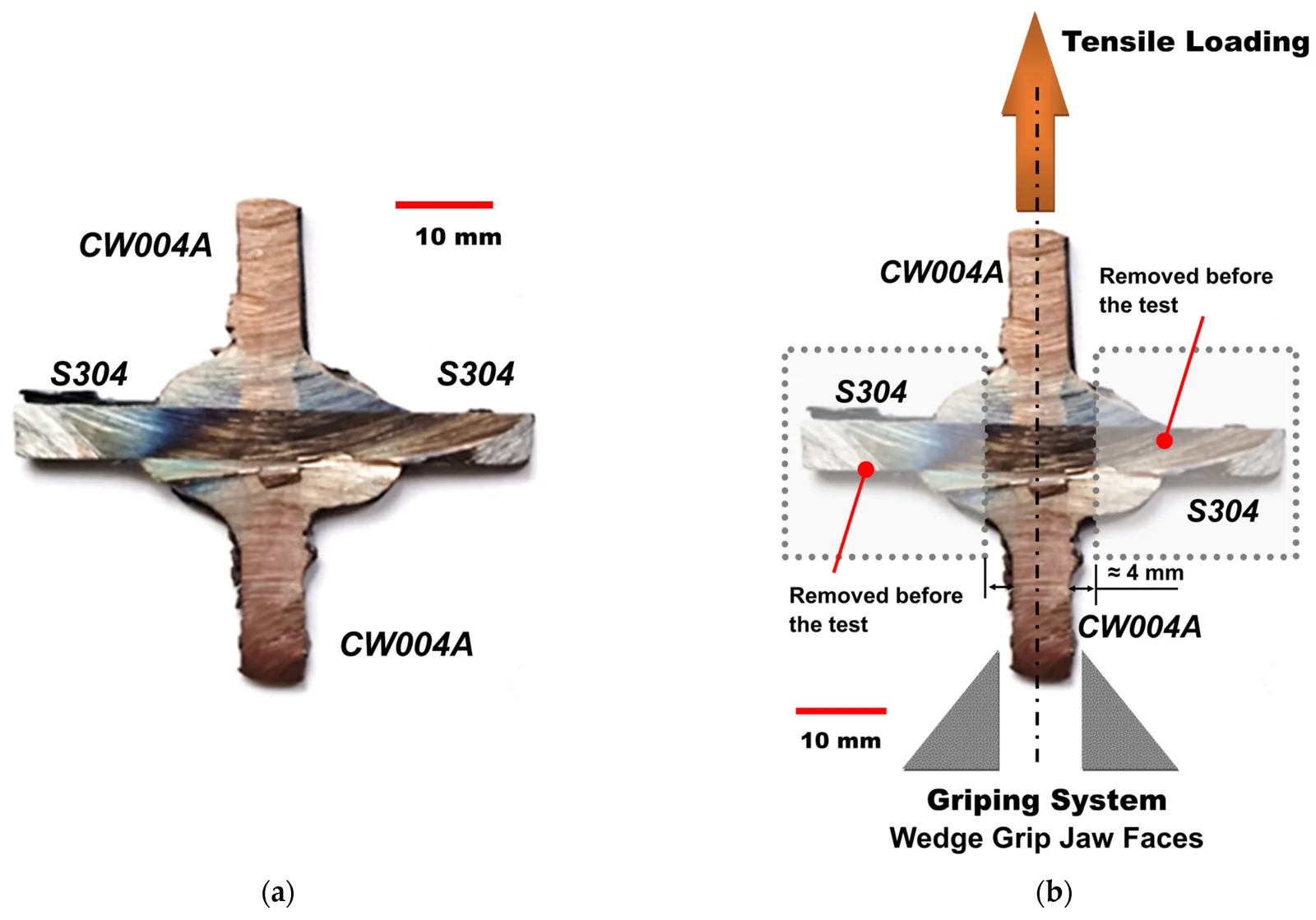
The specimen and tensile test details. (**a**) The manufactured specimen. (**b**) A tensile test scheme, testing machine crosshead speed 5 mm/min.

Cross-type soldered joints were fabricated from 4 mm thick sheets, in which two shorter copper sheets were joined to either side of a vertical austenitic steel sheet (Figure 1). The copper sheets, positioned horizontally, measured 60 mm × 50 mm × 4 mm, while the austenitic steel sheet, positioned vertically, measured 120 mm × 50 mm × 4 mm. The braze joints were deposited as a single layer. High-purity argon was used as the shielding gas, with a flow rate of 13.5 dm^3^/min.

### 2.1. Properties of Base Materials

The mechanical properties of the joined materials are presented in Table 2.

Both materials also have completely different mechanical properties, which makes the brazing process more difficult. Additionally, coupled elements are characterized by different physical materials properties Table 3.

The data presented in Table 2 and Table 3 indicate significant differences in the mechanical and physical properties of copper CW004A and austenitic stainless steel AISI 304. According to the data in Table 2, AISI 304 steel exhibits approximately three times higher ultimate tensile strength and twice the hardness of copper. In contrast, copper has higher electrical conductivity and higher thermal conductivity than AISI 304 steel. These differences in material properties may complicate the conventional fusion welding of the two materials. Therefore, weld brazing was selected as the joining process.

### 2.2. Wires for Braze Welding Processes, Properties

Two various electrode wires containing Sn (CuSn6) and silicon (CuSi3) additions can be used for MIG braze welding (in argon). Table 4 presents information about tested electrode wires.

The table data indicate that all wires have different melting temperatures, which may affect the quality of the welding. A systematic analysis of the literature [24,25,26] shows that CuSi3 wire begins to solidify faster under the same welding conditions, which reduces the time of diffusion processes and the possibility of undesirable phases. Therefore, a brazing wire with added silicon was used in the soldering process. 

An EWM Phoenix 351 welding machine was used to produce the braze-welded joints. To create the joint, an austenitic steel sheet was clamped in a vice, and a fillet joint was made at a 45-degree angle with a copper sheet positioned perpendicularly.

### 2.3. Process Procedure

Before braze welding, surfaces of both steel sheets were carefully cleaned of oxides using brushing and chemical procedures using citric acid (eventually phosphoric acid). Borate- or fluoride-based fluxes were not used, as the process was conducted in a protective atmosphere using argon as the shielding gas in the MIG (metal inert gas) process.

The decision was made to examine the characteristics of braze-welded joints using a silicon-alloyed electrode wire (Table 3) by varying the following parameters:

- The arc voltage was constant at 19 V or 21 V;
- The welding current was varied twice: 150 A and 170 A;
- The braze welding speed was varied twice: 220 mm/min and 320 mm/min.

Braze welding process was performed without preheating.

## 3. Research Methods

### 3.1. Non-Destructive Tests and Statistical Analysis

The braze welding process was carried out without preheating. After welding, non-destructive tests (NDT) were performed. Visual testing (VT) under 750 lx illumination and penetrant testing (PT) were selected for this purpose. Both tests were conducted in accordance with standards EN ISO 17637 [27] and EN ISO 3452-1:2021 [28]. Before application of the penetrant, the examined surfaces were mechanically cleaned to remove loose oxides and surface contamination and subsequently degreased using BRE-S cleaner (DIFFU-THERM, Helmut Klumpf Technische Chemie GmbH & Co. KG, Herten, Germany). The surfaces were allowed to dry completely before penetrant application. BDR-L penetrant was then applied uniformly and allowed to dwell for 30 min. Excess penetrant was removed using a clean lint-free cloth, followed by wiping with a cloth lightly moistened with BRE-S cleaner, taking care not to flush penetrant from surface-breaking discontinuities. After evaporation of the cleaner, a thin and uniform layer of BEA developer was applied. The development time was 30 min.

The examination was conducted at approximately 20–25 °C under white-light illumination of approximately 750 lx. Following development, the surfaces were visually evaluated for relevant linear and non-linear indications. Crack-like linear indications, porosity-related rounded indications, blisters, and other surface-breaking discontinuities were recorded. For the purposes of the present comparative study, a joint was classified as correct when no relevant surface-breaking indications were detected in the examined braze-weld region; samples exhibiting relevant indications were classified as defective according to the type of discontinuity observed.

The NDT parameters for the braze welds (joining method 973) are summarized in Table 5.

A total of 40 samples were prepared for research purposes, (5 samples of each type).

The effects of MIG braze-welding parameters on Cu–AISI 304 joint quality were evaluated using a three-factor analysis of variance (ANOVA) based on a full 2^3^ factorial experimental design. The investigated factors were welding current (150 and 170 A), arc voltage (19 and 21 V), and welding speed (220 and 320 mm/min), each coded at two levels (−1 and +1). Eight experimental combinations were investigated, with five independently fabricated joints per combination, resulting in 40 observations (Table 6). Joint quality was expressed as a binary response, where Y = 1 indicated an acceptable joint and Y = 0 indicated an unacceptable joint based on the adopted NDT assessment criteria. The factorial ANOVA included three main effects, three two-factor interactions, and one three-factor interaction. The effects were determined from differences between the mean responses at the corresponding coded factor levels. The contributions of individual factors and interactions were quantified using their sums of squares relative to the total sum of squares. Due to the binary nature of the response and the violation of conventional ANOVA assumptions, the factorial ANOVA was used primarily for descriptive assessment of effect magnitudes and variance contributions. Statistical significance was additionally assessed using Firth’s [6] bias-reduced binomial logistic regression to account for complete or quasi-complete separation. Individual model terms were evaluated using penalized likelihood-ratio tests, while the joint contribution of interaction terms was assessed using an exact conditional test. The significance level was set at α = 0.05. An exact conditional likelihood-ratio test was used to compare the additive logistic model with the full factorial model, testing the null hypothesis that the interaction terms jointly contribute no additional explanatory information. The conditional null distribution was obtained by enumerating all admissible allocations of the 16 positive NDT outcomes among eight groups of five independently fabricated joints, conditional on the observed total number of positive outcomes and the factor-specific success totals (15 for high current, 6 for high voltage, and 10 for high welding speed). The test statistic was the likelihood-ratio deviance, calculated as twice the difference between the maximized log-likelihoods of the full factorial and additive logistic models. The probabilities of admissible allocations were determined using their corresponding combinatorial weights. Five admissible allocations were identified, with a total weight of 1630. The exact *p*-value was calculated by summing the conditional probabilities of allocations with a test statistic at least as large as the observed value (D = 17.558), yielding *p* = 5/1630 = 0.0031.

### 3.2. Static Tensile Test

Static tensile tests were performed at room temperature using an Instron 3369 (Instron Corporation, Norwood, MA, USA) universal testing machine. Because of the specific geometry of the investigated Cu–austenitic steel cross-joint, non-standard rectangular specimens with dimensions of 50 × 4 × 4 mm were used. The testing procedure was based on EN ISO 6892-1 [29]. The specimens were machined from the braze-welded assemblies so that the braze-welded region was located in the central part of the specimen. The protruding austenitic-steel component was cut to a distance of approximately 4 mm from the specimen face. The specimens were gripped by the copper arms, and the tensile load was applied along the longitudinal axis of the copper part, perpendicular to the original steel component. The nominal load-bearing cross-section was 4 × 4 mm, corresponding to an effective cross-sectional area of 16 mm^2^. Force and displacement were continuously recorded until fracture, and the ultimate tensile strength was calculated as the maximum tensile force divided by the nominal cross-sectional area. The fracture location was documented after testing in relation to the copper base material, braze-welded region, and Cu/filler-metal interface.

### 3.3. Light Microscopy Methodology

Metallographic examination of the joints using a light microscope Axio Observer Z1 (Carl Zeiss Microscopy GmbH, Jena, Germany) was performed in accordance with PN-EN ISO 17639 [30]. The study involved evaluating the material’s microstructure and the quality of the joint. Metallographic specimens were prepared by grinding, polishing (according to the Struers method), and etching in a solution consisting of 15 mL NH_4_OH + 15 mL H_2_O + 5 mL 3% H_2_O_2_ and electrolytical etching of 10% Oxalic acid. In accordance with PN-EN ISO 17639, the microstructure of the weld metal, heat-affected zone, and base material was analyzed, with particular attention to discontinuities such as cracks or porosity, as well as characteristic structural features, including grain boundaries and precipitates.

### 3.4. Scanning Electron Microscopy Methodology

Scanning electron microscopy (SEM) was used to investigate the structure of the samples on Zeiss Supra 35 and Crossbeam 350 (Carl Zeiss AG, Jena, Germany). SEM observations were performed at an accelerating voltage of 5–20 kV using the SE and InLens detector, allowing for high-resolution imaging of surface features with minimal sample damage. Samples were analyzed using orientation imaging microscopy (OIM) with electron backscattered diffraction (EBSD) on a Zeiss Supra 35 SEM (Carl Zeiss AG, Germany). In the energy-dispersive X-ray spectroscopy study the UltraDry detector by Thermo Scientific and Oxford Instruments detector was used (Thermo Fisher Scientific, Waltham, MA, USA, Oxford Instruments, Abingdon, UK). OIM measures crystal orientations on a regular grid through electron diffraction, and the resulting data is used to create orientation or phase maps of the scanned area on the sample. The grain structure was examined by scanning a 100 × 250 µm area on the cross section of the remelting zone with a step size of 0.2 µm. The data was processed using a level 4 neighbor orientation correlation and a grain confidence index standardization with a 2-degree grain tolerance angle threshold.

## 4. Results and Discussion

### 4.1. NDT Test Results

The designations and results of each type of tested sample are given in Table 6.

The results confirmed that even small variations in process parameters significantly affected the quality of the resulting joints. Only three cases produced satisfactory connections. In most samples, defects such as porosity, cracks, or inadequate wettability were observed. These findings highlight the critical importance of proper selection and consistent maintenance of process parameters in determining joint quality. For the purposes of this study, a joint was classified as correct when VT showed continuous and uniform filler-metal spreading and PT revealed no relevant surface-breaking indications. Samples exhibiting insufficient wetting, porosity or gas-related indications, blistering, or linear crack-like indications were classified according to the dominant observed discontinuity. This classification was used as a study-specific comparative criterion rather than as a formal acceptance level according to a welding acceptance standard. Prior to the main analysis, a process map was created to identify the favorable brazing conditions for the materials under study. For this purpose, effective linear energy was calculated, with a process factor of 0.8 applied (Table 7).

**Figure 2:**
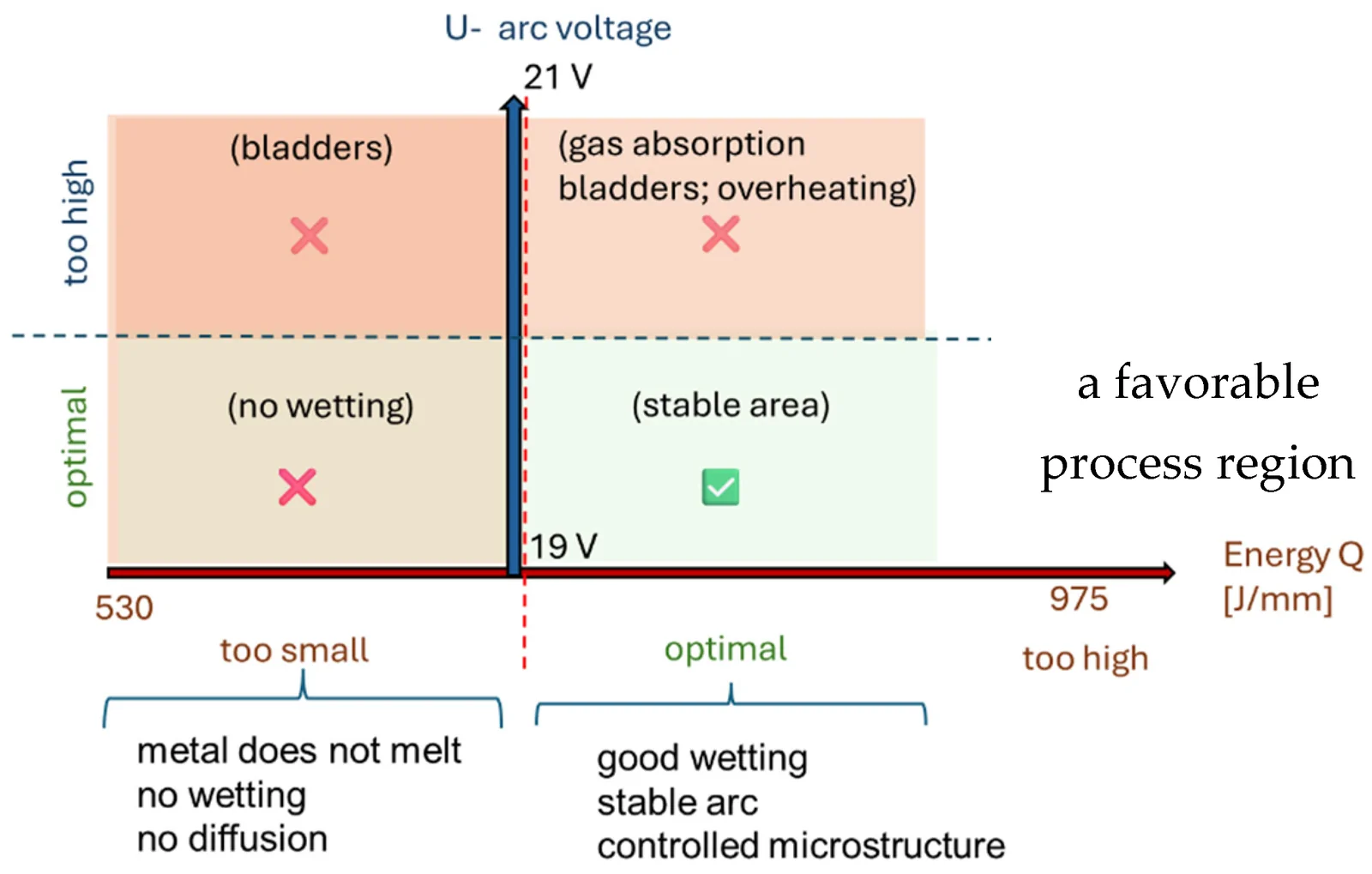
Process map—determination of joint quality.

The results were presented as a graphical process map (Figure 2).

The full 2^3^ factorial model was expressed using a binomial logistic link function:

(1) $$\begin{array}{c} \mathrm{logit}(p)\;=\;\ln(\frac{p}{1\;-\;p})\\ \mathrm{logit}(p)\;=\;\beta_{0}+\beta_{A}A+\beta_{B}B+\beta_{C}C\\ +\beta_{\mathrm{AB}}\mathrm{AB}+\beta_{\mathrm{AC}}\mathrm{AC}+\beta_{\mathrm{BC}}\mathrm{BC}+\beta_{\mathrm{ABC}}\mathrm{ABC} \end{array}$$

where *p* denotes the probability that a joint is classified as acceptable (Y = 1). A, B, and C denote welding current, arc voltage, and welding speed, respectively, coded at −1 and +1. The coefficients β_0_, β_A_, β_B_, and β_C_ describe the intercept and main effects; β_AB_, β_AC_, β_BC_, and β_ABC_ represent the interaction coefficients.

Firth’s bias-reduced logistic regression was used to estimate the coefficients, addressing the separation present in the binary NDT dataset presented in Table 8.

Within the investigated parameter range, the process map identified a favorable process region in which joints meeting the adopted NDT quality criterion were obtained. The results indicated three regions associated with unsatisfactory joint quality and one favorable process region corresponding to parameter combinations producing acceptable joints. Each experimental parameter combination was evaluated using five independently fabricated joints, resulting in a total of 40 NDT observations. All five joints were classified as acceptable under conditions n3, n4, and n8, whereas only one acceptable joint was obtained under condition n5. The remaining parameter combinations did not produce any joints meeting the adopted quality criterion. To quantify the influence of the investigated processing parameters, the main and interaction effects were calculated using the full 2^3^ factorial design. The analysis indicated that welding current exhibited the largest descriptive main effect on joint quality (+0.70), accounting for approximately 51.0% of the total sum of squares of the binary NDT response [6]. Arc voltage and welding speed showed smaller main effects of −0.20 and +0.20, respectively, each contributing approximately 4.2% of the total variability. The interaction effects between welding current and arc voltage, welding current and welding speed, and among all three processing parameters were −0.30, +0.30, and +0.30, respectively, each accounting for approximately 9.4% of the total sum of squares.

Since the response variable was binary (Y = 0 or 1), and seven of the eight experimental groups exhibited no within-group variability, the assumptions of conventional analysis of variance were not satisfied. Therefore, statistical inference was additionally performed using Firth’s bias-reduced binomial logistic regression, including all three main effects, all three two-factor interactions, and the three-factor interaction. Factor levels were coded as −1 and +1, and statistical significance was evaluated using profile penalized likelihood-ratio tests. The logistic regression analysis identified a statistically significant effect of welding current (*p* = 0.0024), whereas the individual main effects of arc voltage (*p* = 0.3864) and welding speed (*p* = 0.3864) were not statistically significant. None of the individual interaction coefficients reached statistical significance (*p* ≥ 0.1287). Nevertheless, an exact conditional test comparing the additive logistic model with the full factorial model demonstrated a statistically significant joint contribution of the interaction terms (*p* = 0.0031). This finding indicates that the relationship between the processing parameters and joint quality cannot be adequately described by their independent main effects alone. However, the limited sample size prevents the reliable identification of individual interaction terms as statistically significant. The statistical significance of the welding current effect and the joint contribution of interaction terms applies exclusively to the investigated parameter ranges and the study-specific binary NDT quality criterion and should not be interpreted as evidence of a universal relationship governing the MIG braze-welding process.

This interaction-dependent behavior is particularly evident when comparing conditions n7 and n8. At identical welding current (170 A) and arc voltage (21 V), increasing the welding speed from 220 to 320 mm/min was associated with an increase in the proportion of acceptable joints from 0% to 100%. These findings support the interpretation of the process map in terms of combined processing conditions rather than isolated parameter effects. The constructed process map also provides insight into the relationship between linear energy and joint quality. The experimental results suggest that joint quality depends on the combined processing conditions rather than on a single optimal linear energy value. As shown in Table 7, the effective linear energies corresponding to conditions n3, n4, and n8, which produced exclusively acceptable joints, were approximately 704.73, 484.50, and 535.50 J/mm, respectively. In contrast, condition n5, characterized by an effective linear energy of approximately 687.27 J/mm, produced only one acceptable joint out of five, while condition n7, with an effective linear energy of approximately 778.91 J/mm, resulted in no acceptable joints. These observations indicate that an effective linear energy close to 700 J/mm does not, by itself, guarantee satisfactory joint quality. The effective linear energy was calculated using a thermal efficiency factor of η = 0.8, adopted for the MIG process according to EN 1011-1:2009 [31]. Accordingly, the calculated effective linear energy corresponds to 80% of the nominal arc energy. This coefficient represents an assumed process efficiency rather than an experimentally determined value of the actual heat transferred to the workpiece.

Overall, the factorial analysis and bias-reduced logistic regression support the experimental observations presented in the process map and identify welding current as the dominant factor associated with joint quality within the investigated parameter range. The statistically significant joint contribution of the interaction terms further demonstrates that the combined effects of welding current, arc voltage, and welding speed should be considered when selecting suitable processing conditions. Nevertheless, the limited number of investigated parameter levels, the relatively small sample size, and the binary nature of the NDT classification restrict the generalization of the identified favorable processing region. Consequently, the proposed process window should be regarded as specific to the experimental conditions and comparative NDT quality criterion adopted in this study, rather than as a universally applicable optimization of the MIG braze-welding process.

### 4.2. NDTsts—Analysis and Discussion

Analysis of the results presented in Table 6 indicates that the selected welding current plays a critical role in the brazing process. In most cases, higher current values (170 A) improved the quality of the resulting joints (samples N3, N4, N8). When lower current values (150 A) were applied, the joints exhibited welding defects. For example, it was observed that the filler metal did not spread evenly over the surface of the austenitic steel (samples N1, N2). This indicates that at low current, the process temperature is insufficient to overcome surface tension, resulting in inadequate wetting of the metal surface. It can be concluded that too low a current leads to incomplete melting of the filler material and reduced wettability, which may cause discontinuities (e.g., cracks in sample N6), ultimately reducing the joint strength in mechanical tests. This observation is consistent with findings reported in the literature [32,33,34].

The results indicate that higher current is beneficial to the process, as it is associated with a higher frequency of correctly formed connectors under the tested conditions. However, connector correctness also depends on other parameters.

Our study highlighted the governing role of higher currents in the brazing process. Such currents provide greater “process robustness,” making the joint quality less sensitive to changes in other parameters.

An increase in current leads to a greater amount of molten filler material and more intense heating of the base material. As a result, the experiments showed increased penetration depth and an expanded heat-affected zone. These observations are consistent with reports in the literature [33,34], Excessively high current can lead to overheating of the material, deformation, and damage. In our study, no such defects were observed, indicating that the current range selected for the process was appropriate. Another critical factor influencing the quality of the joint is the arc voltage. If improperly selected, excessively high voltage increases the risk of gas-related defects within the joint. NDT examinations of samples N5 and N7 revealed discontinuities in the form of gas porosity. Arc voltage primarily affects the arc length and the metal transfer characteristics within the arc. Higher voltage produces a wider arc, which increases the volume of molten metal and promotes greater gas absorption (as illustrated in the process map, Figure 2). High arc voltage combined with low brazing current favors gas entrapment within the solder joint [35,36]. The brazing process conducted at low current values and high arc voltage is characterized by a lower joining temperature compared to that generated at higher current intensities. Consequently, this results in a deterioration of the degassing process. Conversely, a higher process temperature (at increased current intensities) may, in certain cases, compensate for the adverse effects of elevated voltage, as observed in sample n8.

In the majority of samples produced at an arc voltage of 21 V, porosity was identified, whereas no such discontinuities were detected in samples fabricated at 19 V. It was also observed that higher arc energy may intensify thermal stresses (as exemplified by sample n6, where cracking at the Cu–binder interface was detected). Thus, it can be concluded that excessively high voltage leads not only to reduced process stability and increased metal spatter, but above all to the formation of various types of discontinuities within the joint. For the investigated materials, an arc voltage of 21 V can be considered excessively high. According to literature data [35,36,37,38], Excessively low voltage in welding processes leads to arc shortening and may result in process instability, limited wettability of the base material, and the formation of a narrow weld with unfavorable geometry. Such imperfections were not observed in the conducted experiments; therefore, a voltage of 19 V can be considered appropriate. Another important parameter is the brazing speed, which determines the duration of thermal interaction with the base material. As the torch travel speed increases, the heat input to the joint decreases, resulting in a reduction in weld width and a smaller heat-affected zone. Thus, a lower brazing speed (220 mm/min) promotes better joint penetration, as it allows more time for heat transfer. No imperfections were observed in joint n3. An interesting observation is that at high welding current, the process speed becomes less significant. Both samples n3 and n4 exhibited good joint quality, despite being produced at different travel speeds. In these cases, the welding current and arc intensity were the dominant factors governing joint quality. In contrast, the analysis of sample n2 indicates that a higher process speed (320 mm/min) may deteriorate wettability under low current conditions. The reduced joint quality in this case results from insufficient thermal interaction time, as confirmed by findings reported in the literature [34,35,36,37,38,39].

The appropriate selection of arc voltage, current intensity, and brazing speed is of critical importance for obtaining a joint with proper geometry, good wettability, and high mechanical performance. Optimization of these parameters enables the reduction in the heat-affected zone, minimizes the risk of welding defects, and ensures high quality and durability of the braze-welded joint. A summary of the metallurgical mechanisms responsible for the formation of imperfections in the joints is presented in Table 9.

A detailed analysis of the NDT results, with respect to the underlying metallurgical mechanisms, confirms that the proper selection of the process linear energy is important for obtaining high-quality samples, but it cannot serve as the sole criterion determining joint properties. The results demonstrated that, at similar linear energy levels, it is possible to obtain both defect-free materials and materials exhibiting welding imperfections. The analysis showed that different combinations of arc voltage, current intensity, and process speed produce distinct physical effects, which in turn affect joint quality. While the linear energy of the process can serve as a technological criterion for sample fabrication, the quality optimum depends not only on the linear energy but also on the manner in which it is delivered. To assess how variations in brazing parameters influence the mechanical performance of the joints, tensile tests were conducted. Only correctly fabricated joints (n3, n4, n8) were included in the study. The purpose of these tests was to verify whether a quality optimum determined solely based on NDT results is sufficient to achieve optimal mechanical properties.

### 4.3. Static Tensile Test Results

**Figure 3:**
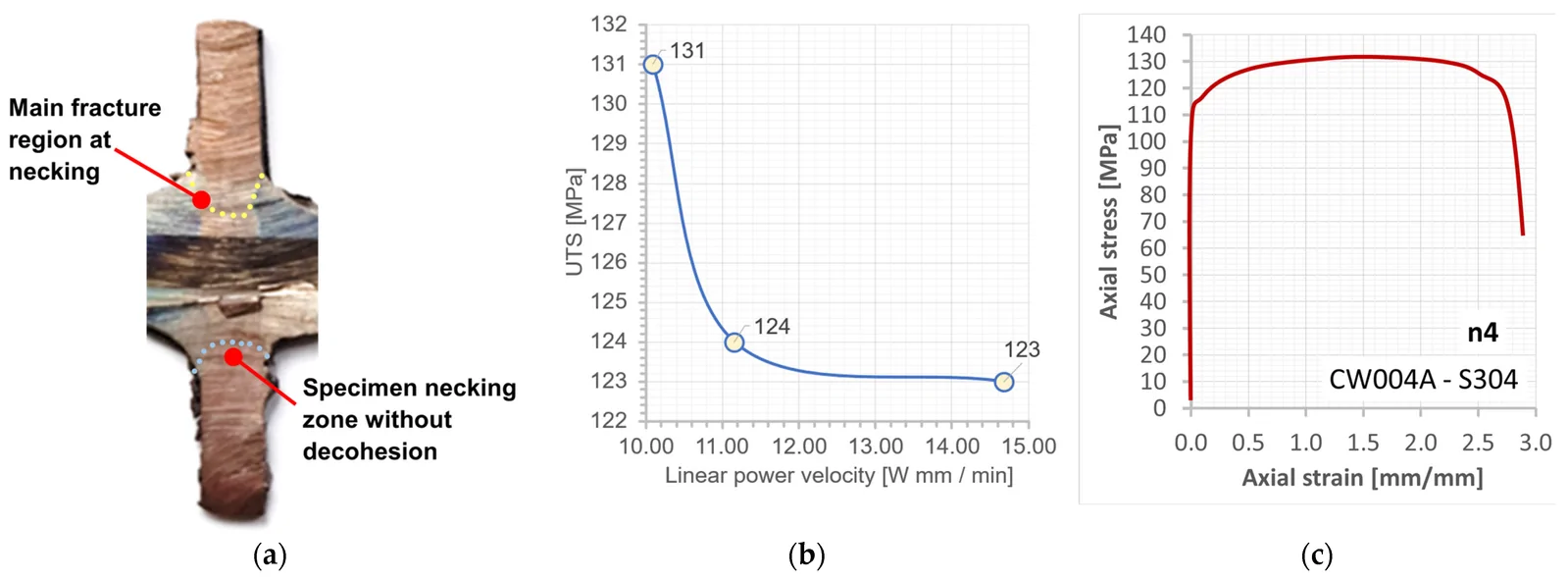
The joint after tensile test, (**a**) damage zones and their features (**b**), and static tensile test curve (**c**).

The strength results of braze welded joints are presented in Figure 3 and Table 9.

The results presented in Figure 3 and Table 10 indicate comparable mechanical properties of the joints. The tensile strength range of the tested samples is relatively narrow, approximately 6%. This suggests that brazing process parameters falling within the so-called quality window, as defined by the process map in Figure 2, ensure process stability when joining copper to austenitic steel. Nevertheless, the observed differences in the strength of individual samples require detailed analysis to determine the optimal process conditions for achieving joints with the best mechanical performance.

As can be seen, the UTS values decreased to 123.5 MPa when linear power energy was applied to the welding process. However, this could be considered an undesirable mechanical parameter, given that higher values were observed at a welding power of 10.5 W/mm/min. This could be significant for determining joint quality. Nevertheless, despite the differences in UTS, no changes to the joint were observed. The main fracture with necking occurred in the upper part of the joint, as shown in Figure 3a.

The differences between the three experimental groups were evaluated using one-way analysis of variance (ANOVA), followed by Tukey’s honestly significant difference (HSD) post hoc test. The homogeneity of variances was evaluated using Levene’s test, while the normality of residuals was assessed using the Shapiro–Wilk test. No statistically significant deviations from these assumptions were detected (Levene’s test: *p* = 1.000; Shapiro–Wilk test: *p* = 0.216), although the small sample size limits the sensitivity of these tests. The ANOVA revealed statistically significant differences in mean tensile strength among the investigated configurations (F(2, 12) = 127.52, *p* = 8.23 × 10^−9^). The effect size, expressed as eta-squared (η^2^ = 0.955), indicated that approximately 95.5% of the total observed variation in UTS within the three selected experimental groups was associated with differences between the group means. This effect size applies only to the investigated configurations and should not be interpreted as the contribution of individual welding parameters across the complete factorial design. Post hoc comparisons using Tukey’s HSD test demonstrated that configuration n4 exhibited significantly higher tensile strength than both n3 and n8. The mean difference between n4 and n3 was 7.60 MPa (95% simultaneous confidence interval: 6.19–9.01 MPa; adjusted *p* = 1.79 × 10^−8^), whereas the difference between n4 and n8 was 7.00 MPa (95% simultaneous confidence interval: 5.59–8.41 MPa; adjusted *p* = 4.55 × 10^−8^). In contrast, the difference between n8 and n3 was only 0.60 MPa and was not statistically significant (95% simultaneous confidence interval: −0.81 to 2.01 MPa; adjusted *p* = 0.513). An additional correction for multiple comparisons using Holm’s procedure confirmed the same pattern of statistical significance. The results of the ANOVA pair test are presented in Table 11.

The comparison between configurations n3 and n4 is particularly relevant because both conditions employed the same welding current (170 A) and arc voltage (19 V), differing only in welding speed. Increasing the welding speed from 220 to 320 mm/min was associated with an increase in mean UTS of 7.60 MPa, corresponding to approximately 6.2% relative to n3. Similarly, configurations n4 and n8 were produced at the same welding current (170 A) and welding speed (320 mm/min), differing only in arc voltage. Reducing the voltage from 21 to 19 V was associated with a 7.00 MPa increase in mean tensile strength, corresponding to approximately 5.7% relative to n8. These results suggest that both welding speed and arc voltage influence the mechanical performance of Cu–AISI 304 braze-welded joints within the selected processing conditions. However, these comparisons do not establish the independent main effects of these parameters across the full factorial design. Furthermore, the results indicate that configuration n4, characterized by an effective linear energy of approximately 484.50 J/mm, provided the highest tensile strength among the three investigated configurations. Nevertheless, this observation does not establish a universal relationship between decreasing linear energy and increasing mechanical strength, since the tensile tests were restricted to configurations previously classified as acceptable according to the NDT criterion.

### 4.4. Static Tensile Test—Analysis and Discussion

The analysis of the results presented in Table 9 indicates that the highest tensile strength was achieved for the highest welding speed (sample n4). It is likely that the increased welding speed and faster cooling limited dendritic grain growth and suppressed excessive formation of brittle phases. The resulting microstructure provided higher strength compared to that formed at lower welding speeds. According to literature data [40], shorter thermal exposure time restricts grain growth, which in our case explains the higher strength values obtained for samples n4 and n8 compared to sample n3. Furthermore, as noted in [41], excessively low process speeds can reduce joint strength, for example due to overheating, grain coarsening, and the potential formation of brittle phases. The lowest UTS was observed in sample n3. Notably, increasing the voltage from 19 V to 21 V led to a reduction in tensile strength. For sample n4, the strength was 131 MPa, whereas for sample n8 it was 124 MPa. It is known that a wider arc promotes greater material dilution. Previous investigations presented in the preceding chapter demonstrated a correlation between joint porosity and arc voltage. Therefore, it can be assumed that the recorded decrease in strength is associated with a similar metallurgical mechanism. Micropores, likely present in the microstructure of the sample, may be undetectable by NDT but can contribute to a slight reduction in joint strength. All tested samples exhibited similar tensile strength values (123–131 MPa), confirming that the selected brazing current minimizes variations in sample quality, while differences in achieved strength result from the chosen voltage and process speed parameters. The strength range of the tested samples is relatively narrow, approximately 6%, indicating that the selected process parameters ensure both stability and reproducibility of copper–austenitic steel cross joints using a silicon-containing filler metal. The analysis further shows that the best properties are obtained at lower linear energy (Q) (Table 3), which plays a significant role in shaping the joint microstructure, consistent with information reported in [42]. The study clearly demonstrates that quality control alone is insufficient; correct joints made from the same material may exhibit different mechanical properties. The mechanical optimum for the investigated process corresponds to a combination of high welding speed and moderate voltage. This optimum does not coincide with the quality optimum defined solely by NDT, which is typically the focus in scientific publications (acceptable/unacceptable joints). The absence of detected defects does not guarantee optimal joint performance. The best results were obtained for joint n4, produced under the following parameters: I = 170 A, U = 19 V, and V = 320 mm/min.

### 4.5. Microscopic Observation from the Austenitic Side—Results and Analysis

**Figure 4:**
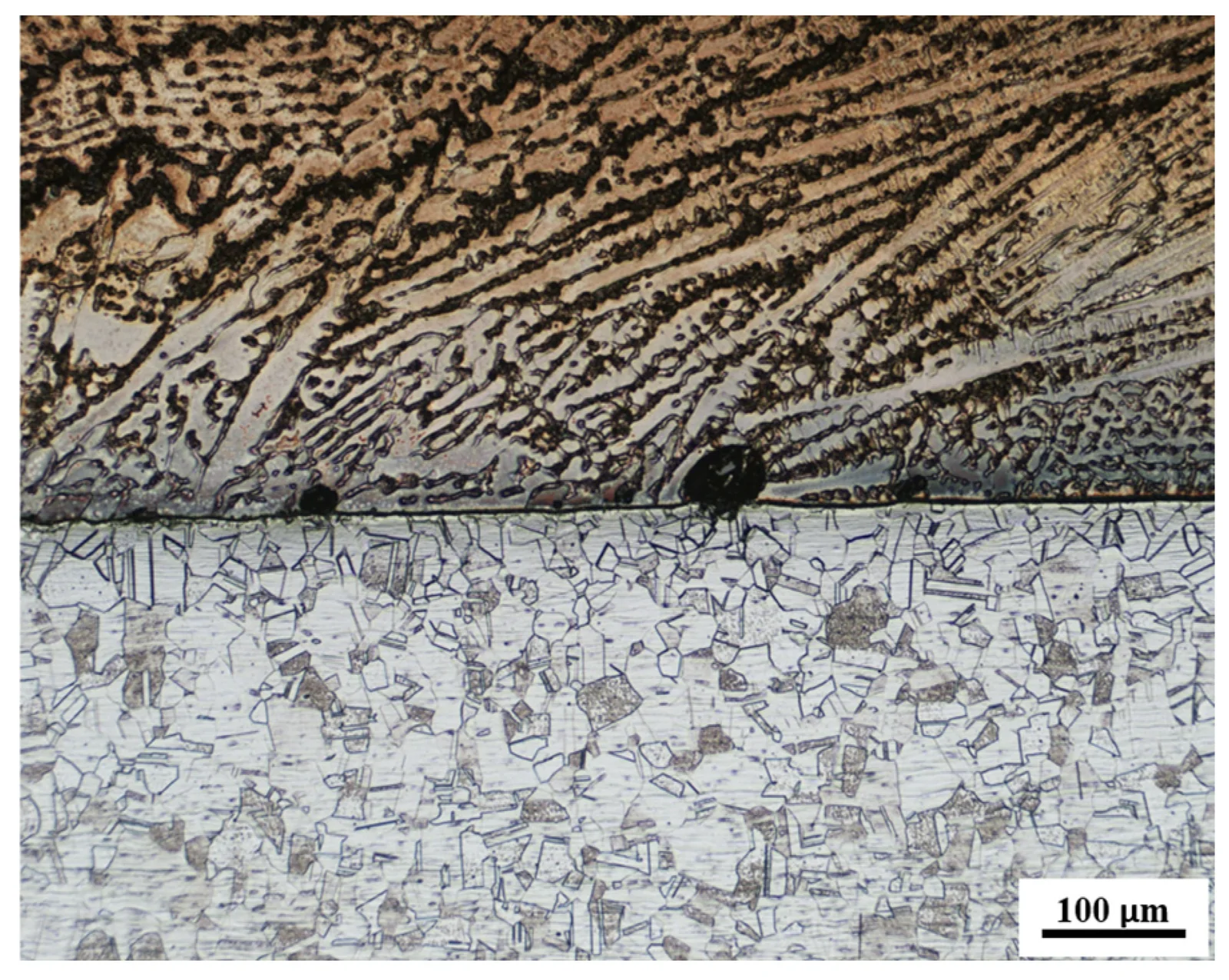
Braze-welded joint using CuSi3 filler wire (austenitic steel, brazed filler)—Etching: 15 mL NH_4_OH + 15 mL H_2_O + 5 mL 3%H_2_O_2_.

Light microscopic (LM) observations were conducted. The joint shown in Figure 4 exhibits a two-zone, multiphase character. The analyzed microstructure reveals two clearly distinct regions: the base material—austenitic steel—and the brazed filler—bronze, separated by a linear interface. This corresponds to the typical morphology of a braze-welded joint between austenitic steel and a copper-based alloy [43].

At the bottom of the photograph (Figure 4), austenitic grains with varied crystallographic orientations can be observed, while at the top, the brazed filler metal is visible, etched in an NH_4_OH-based solution. The observed joint morphology is typical for this type of connection and is characterized by a sharp, well-defined interface between the base material and the filler metal. This boundary shows no visible dendrites extending into the base material, suggesting incomplete melting of the steel.

**Figure 5:**
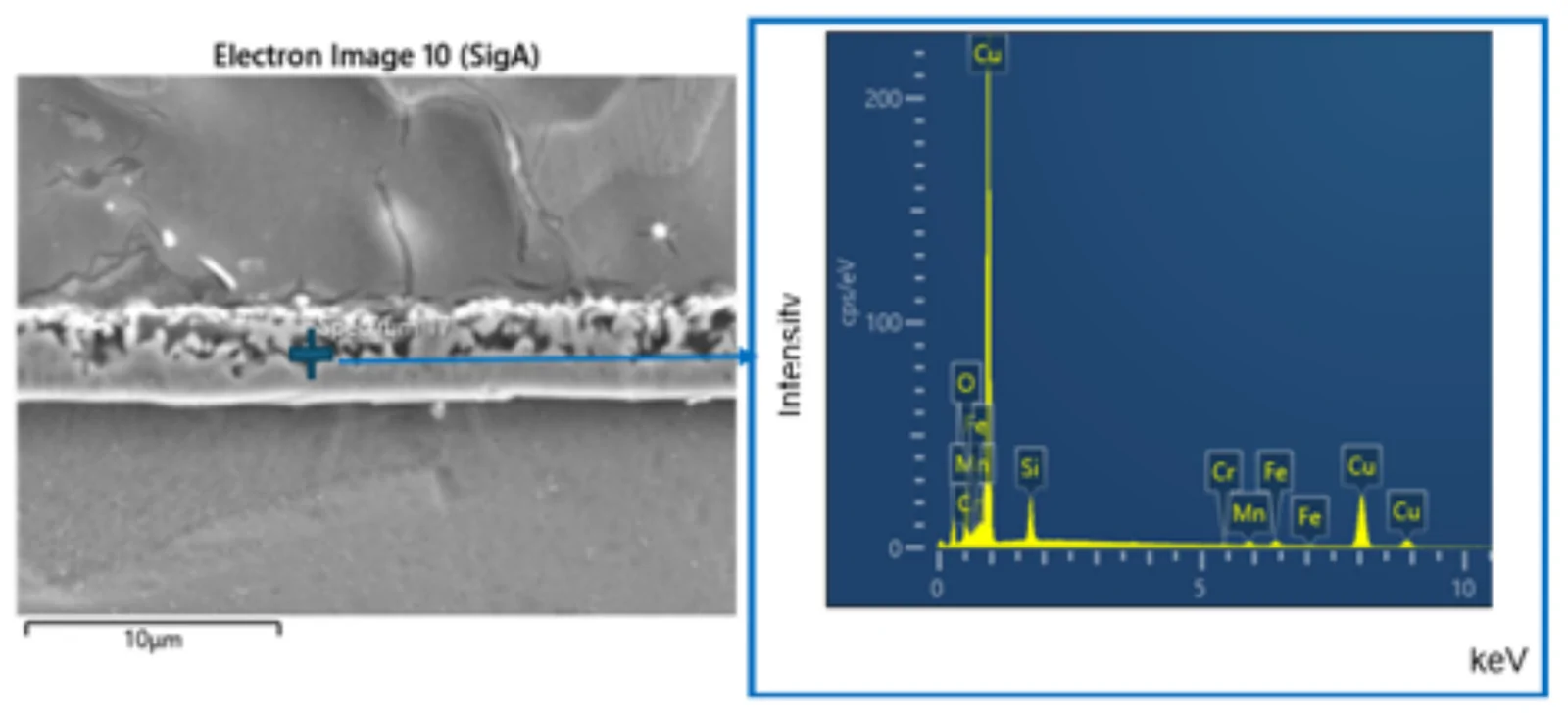
SEM—microstructure and EDS analysis.

Furthermore, based on the Fe–Cu system [44], which exhibits limited mutual solubility, the interface between the materials remains distinct (Figure 4—LM, Figure 5—SEM). The authors’ findings are consistent with the phase diagrams presented in Springer publications.

Changing the magnification during the microstructural analysis of the joint allowed for a more detailed observation of the reaction zone at the interface. In the immediate vicinity of the boundary, a thin layer with distinct contrast was observed, interpreted in the literature as the Fe–Cu–Si diffusion zone. Such layers are typically a few micrometers thick, as described, for example, in [45].

At the top of the photograph (Figure 4), within the microstructure of the joint’s weld metal, a dendritic structure can be observed, which is typical for alloys solidifying from the melt [46]. Similar α-Cu dendrite morphologies have been described in [47]. It should be noted that the observed dendrites are elongated from the joint interface toward the center of the brazed filler, consistent with the direction of the temperature gradient during solidification. This dendrite orientation is further supported by studies on alloy solidification [48].

Micro-area analysis revealed the primary dendrite arms and their cellular structure, with a characteristic size of approximately 10–30 µm (Figure 3). These values should be considered approximate estimates to illustrating the characteristic microstructural features rather than as statistically representative measurements to the qualitative interpretation of the observed microstructural features. The observed variation in dendrite size moving away from the joint interface indicates a change in cooling rate during solidification, as supported by solidification models [46,48]. Contrast differences between dendrite cores and interdendritic regions (Figure 4) indicate microsegregation of silicon and manganese during solidification [49]. At this stage of the study, it can also be inferred that the interdendritic regions may contain silicon-enriched eutectic Cu–Si areas, as demonstrated in SEM/EDS analyses reported, among others, in [50].

In this context, the SEM investigations of the joint will be presented later in the article. Observation of the microstructure of the braze-welded joint from the austenitic steel side indicates that the dominant phase is the α-Cu solid solution, which is confirmed by diffraction studies of CuSi3 filler metal reported in welding and metallurgical literature, e.g., [51,52]. In the examined samples (Figure 3 and Figure 4), the interface between the steel and the brazed filler is almost linear and continuous, indicating stable wetting of the steel surface by the molten Cu–Si alloy. The interfacial region was additionally quantified at five positions for each investigated sample. For the three samples analyzed by SEM, 15 measurements gave an overall thickness of 1.67 ± 0.11 µm. The measurements confirms that the interfacial layer is thin, continuous and exhibits a certain degree of local variation along the joint. The influence of wettability on the shape of the interface formed during brazing has been discussed, among others, in [53]. A detailed review of the literature in this area suggests that the mechanisms governing phase boundary formation, which depend on wetting parameters, can be considered largely universal for brazing processes. Similar principles are presented in [54].

### 4.6. Proposed Model and Mechanism of Joint Formation from the Base Material Side—Austenitic Steel

The detailed analysis of the structural study results presented in the previous chapter allows the authors to propose the following mechanism for joint formation from the base material side—austenitic steel.

In the first stage, the CuSi3 filler wire melts and wets the steel surface. In the next stage, diffusion of elements from the steel into the molten metal can be observed. The final stage involves the formation of a dendritic structure as heat is removed and solidification occurs. The dendritic morphology suggests directional solidification, typical of metal deposition in arc-based processes. Similar mechanisms, although for different joint types, have been described in the literature [55,56,57].

In the microstructures of samples 3 and 4, no visible continuous metallurgical cracks were observed along the joint interface, indicating proper wetting and the absence of continuous brittle phases at the interface. However, literature reports indicate that a thin layer containing Fe–Si and Fe–Cu may form in such joints, usually with dimensions below a few micrometers.

**Figure 6:**
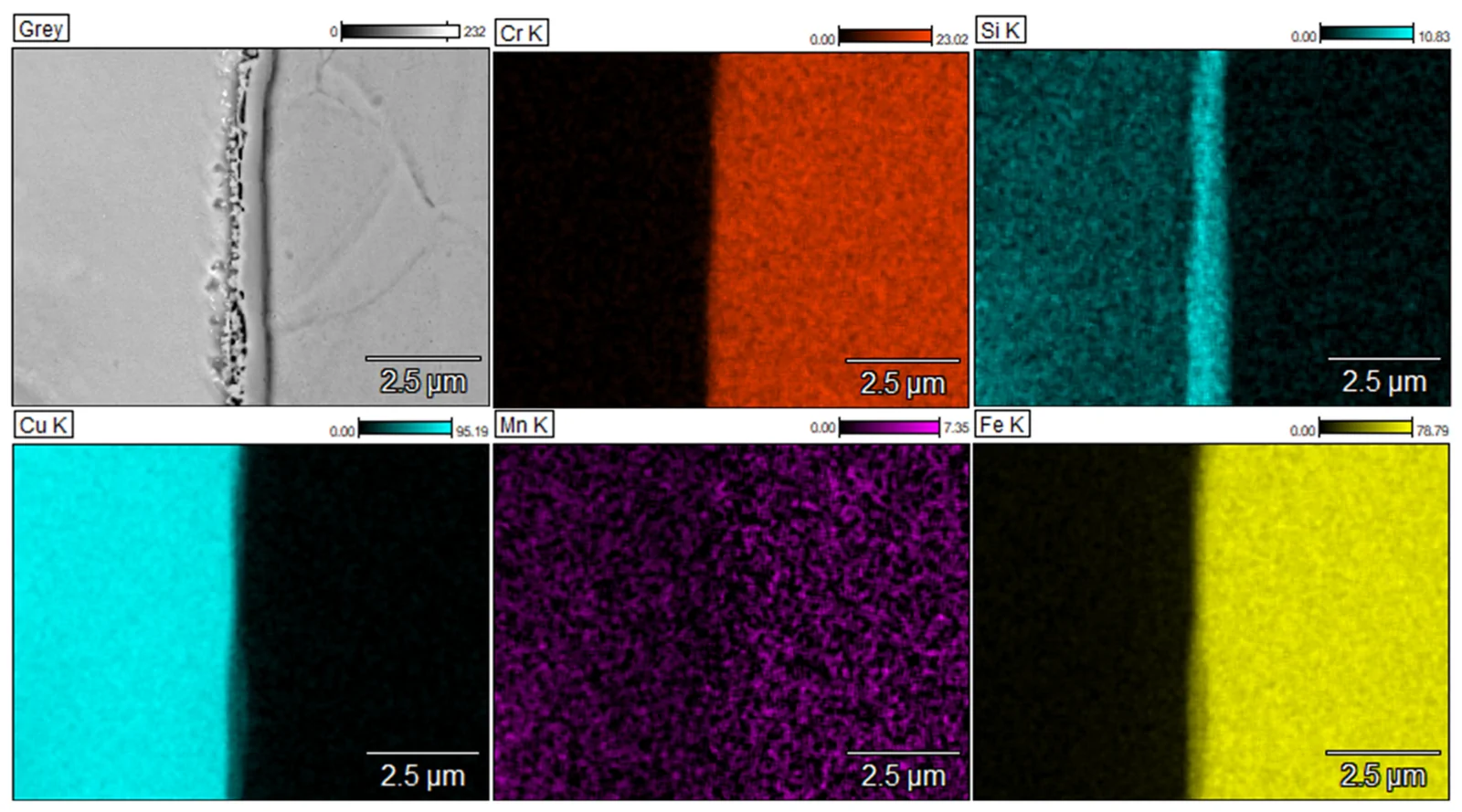
Microstructure of interface (SEM) and EDS map identification of Si, Fe, Mn, Cr and Cu elements in the interface, the contrast (pointers placed and presented photos) corresponds to a greater amount of each element.

A more detailed microstructural analysis was performed using SEM techniques. The analysis confirmed the concentration of Si and Fe at the phase boundary (Figure 6).

**Figure 7:**
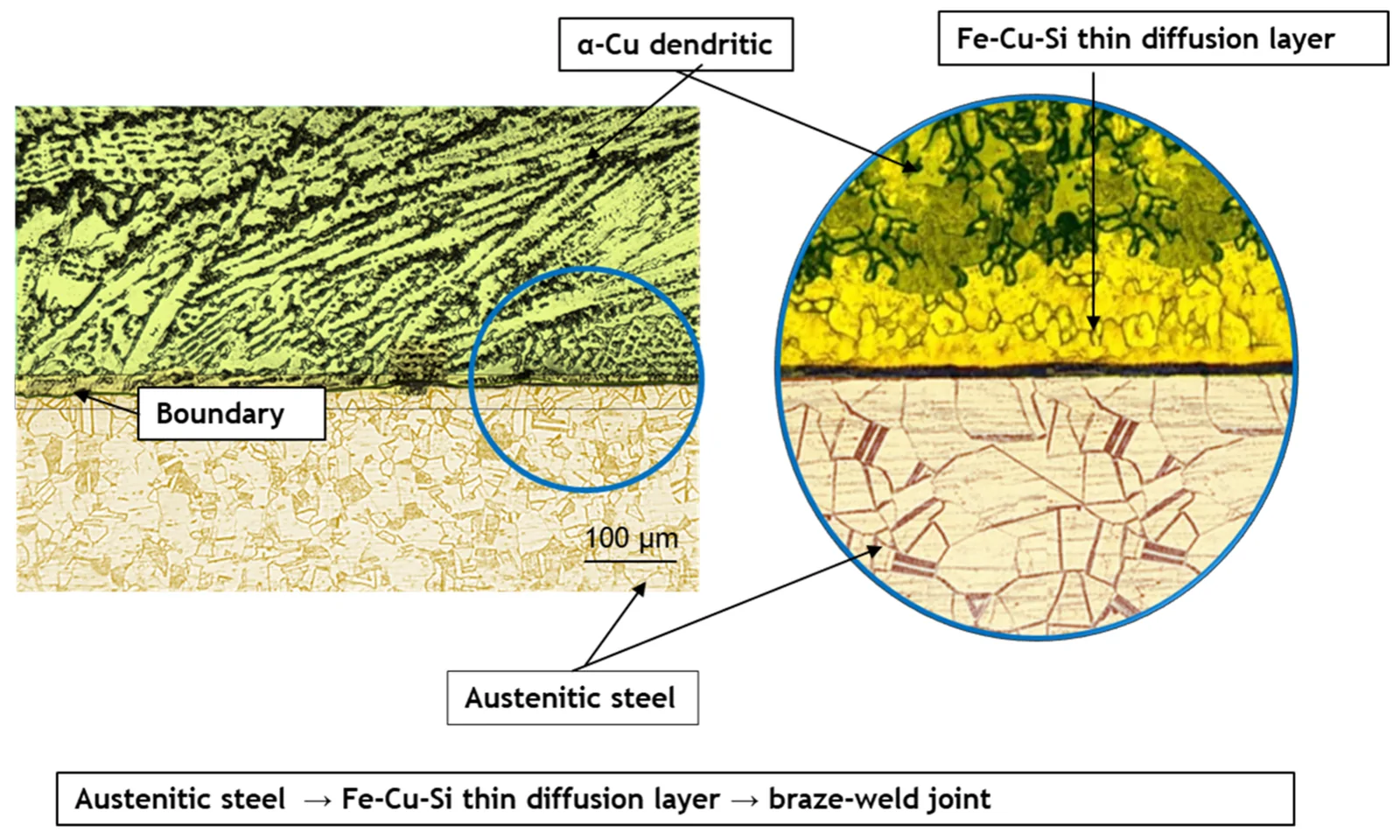
Schematic of the austenite–copper joint, viewed from the austenitic steel side.

CuSi3 brazing is most commonly performed as MIG brazing (GMA brazing), a process intermediate between welding and brazing. The filler metal’s melting temperature is lower than that of the base material. Therefore, the conducted observations allowed identification of a metallurgical joint in which the base material remained unmelted, while the molten filler wetted the steel surface and diffused into the interfacial zone. The observed joint morphology (Figure 3 and Figure 4) corresponds to the following probably microstructural model (Figure 7): austenitic steel → thin Fe–Cu–Si diffusion layer → dendritic α-Cu deposited metal.

Microstructural analysis also indicates that the addition of silicon improved the wettability and fluidity of the filler metal. Furthermore, the use of the brazing process results in a significantly smaller heat-affected zone (HAZ) compared to, for example, arc welding. The reduced HAZ minimizes the risk of deformation during service.

The CuSi3 braze-welded joint represents a diffusion-based metallurgical connection with a Cu–Si solid solution structure, characterized by good steel wettability, a limited heat-affected zone, and moderate mechanical strength, making it particularly suitable for joining thin sheets.

In the subsequent study, the analysis focused on the cross-joint between austenitic steel and copper from the copper side.

### 4.7. Microscopic Observation from the Copper Side—Analysis, Model, and Mechanism

**Figure 8:**
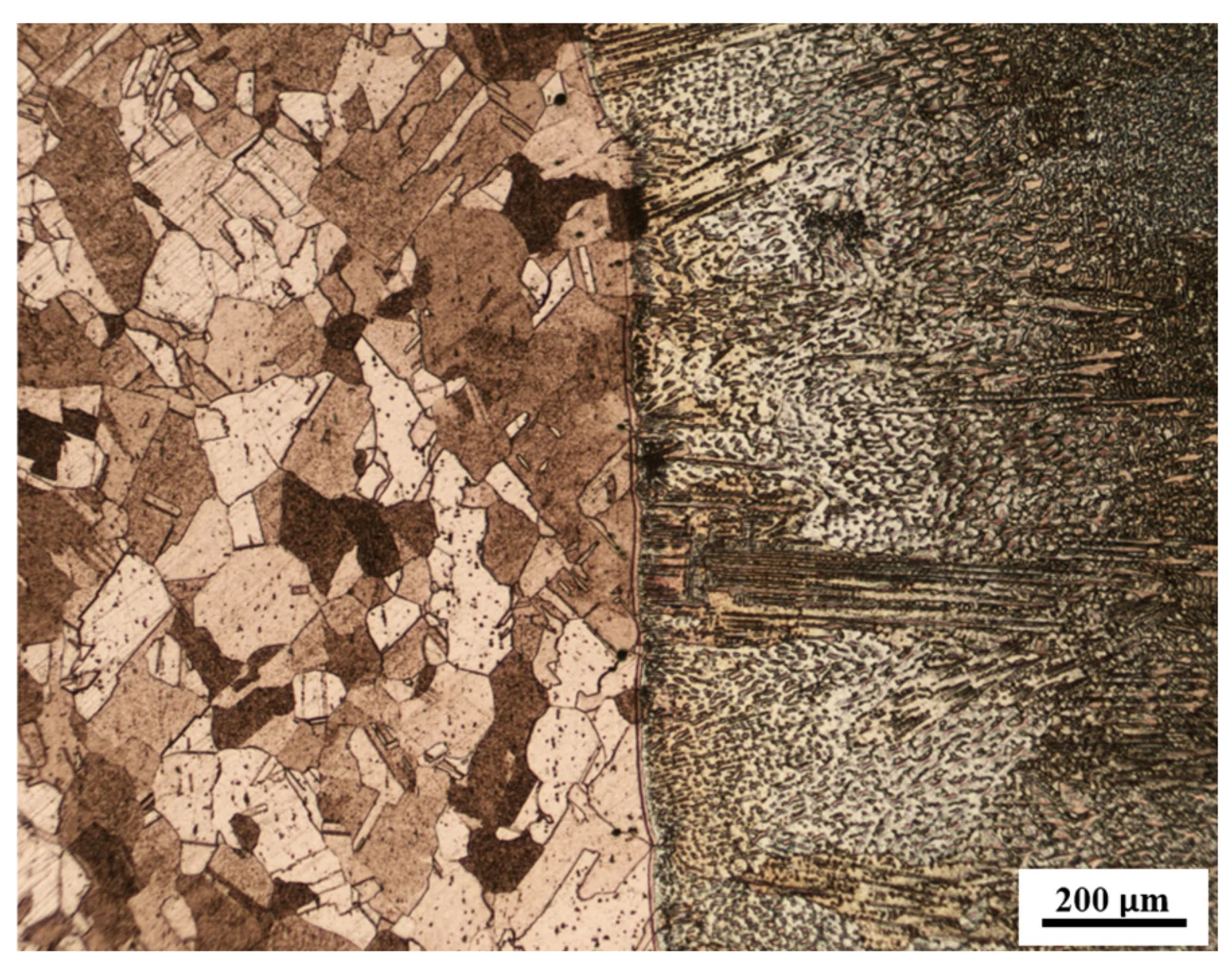
Brazed austenite–copper joint using CuSi3 filler wire—copper side (filler metal region); etching: 15 mL NH_4_OH + 15 mL H_2_O + 5 mL 3%H_2_O_2_.

**Figure 9:**
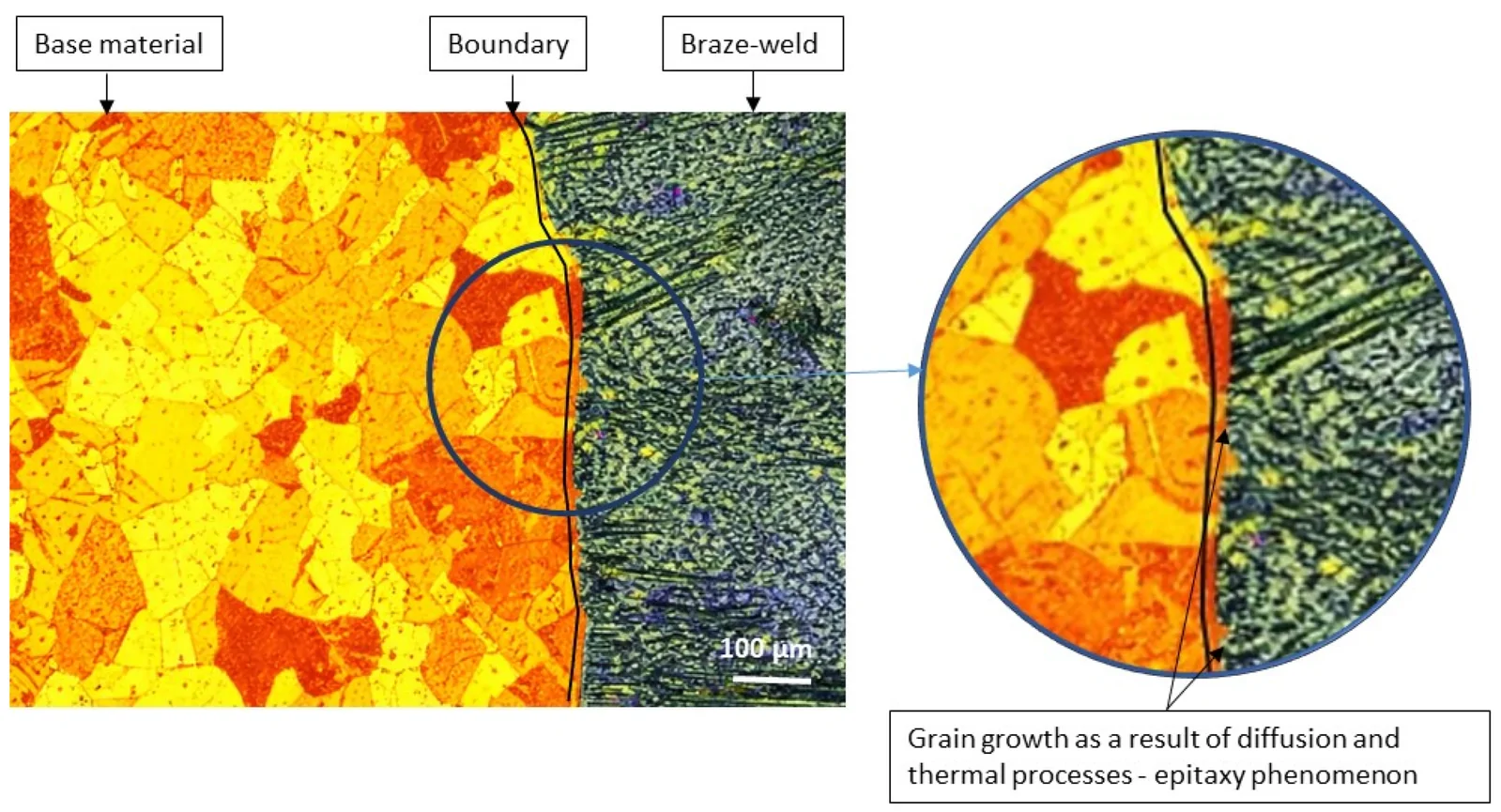
Schematic of the joint highlighting the region characteristic of possible orientation-related grain growth.

Light microscopy observations of the joint from the copper side revealed an apparent continuity of grain morphology across the interface, as shown in Figure 8. This observation may suggest orientation-related interfacial growth; however, epitaxial growth cannot be conclusively confirmed without quantitative crystallographic orientation analysis. A schematic representation of the proposed mechanism is presented in Figure 9.

The microstructural observations indicate an apparent continuity of grain morphology across the joint interface. This feature may be consistent with orientation-dependent grain growth; however, confirming such a mechanism would re-quire quantitative crystallographic analysis, for example, using EBSD. A schematic representation of the joint microstructure is presented in Figure 9.

Global studies [58,59,60,61,62] indicate that grain growth can extend across the joint interface during diffusive and thermal processes, including brazing. For the analyzed structure, and in conjunction with literature on brazing and diffusion joints, the following six-step mechanism of joint formation from the copper side can be proposed:

Step 1 Wetting and crystallographic contact: The molten filler (Cu–Si) wets the surface of the base material, establishing a continuous atomic contact at the phase boundary.

Step 2 Interfacial diffusion: Mutual diffusion of elements occurs, particularly along grain boundaries, which act as preferential diffusion paths during brazing and soldering processes.

Step 3 Heterogeneous nucleation: This step largely determines the morphology of the developing joint. During solidification, nuclei form on the crystalline substrate, with orientations energetically favored relative to the substrate.

**Figure 10:**
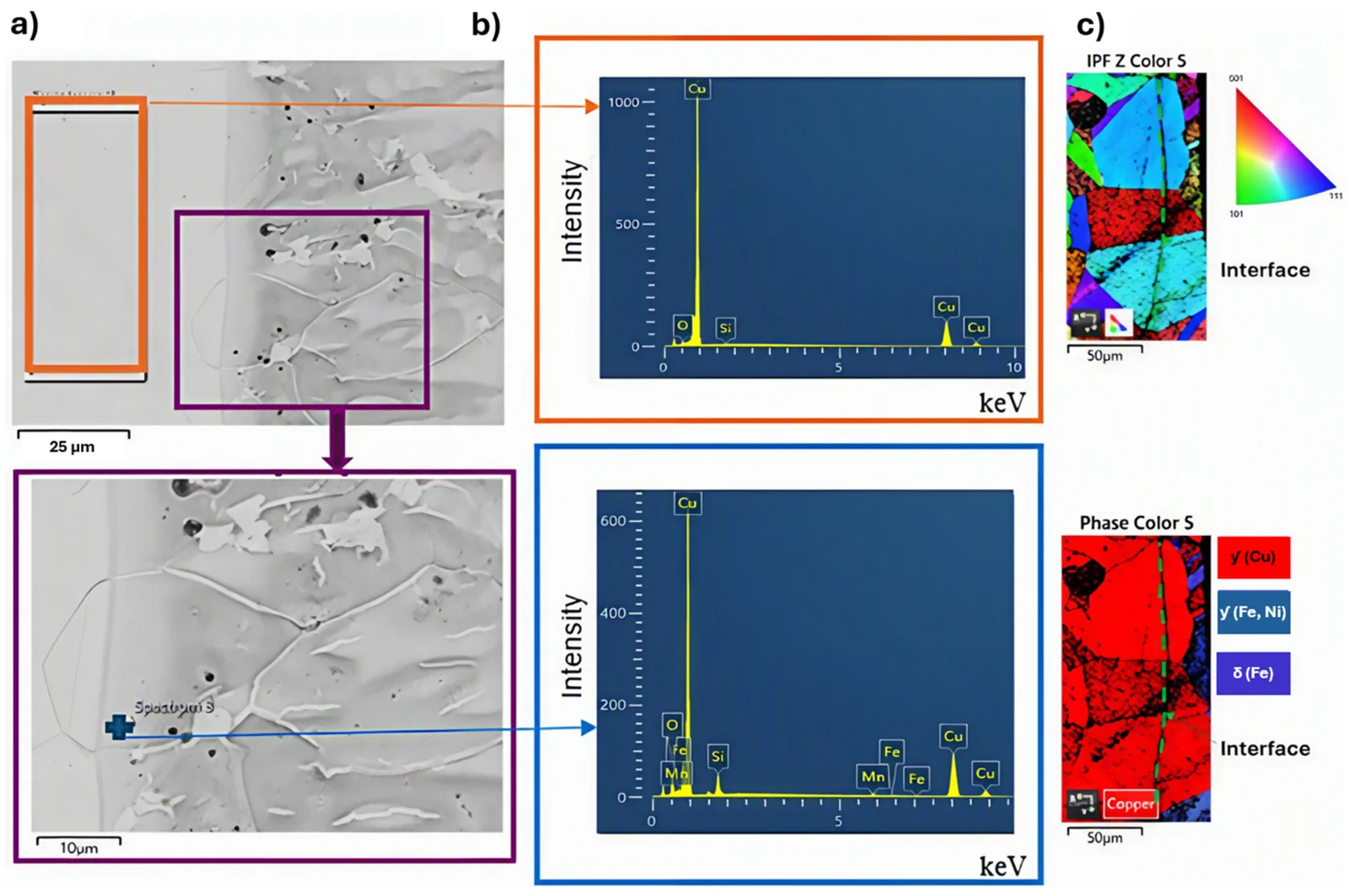
Results: (**a**) SEM, (**b**) EDS and (**c**) EBSD analysis.

Step 4 Possible orientation-related growth: During solidification, filler-metal grains may develop crystallographic relationships with the substrate. The apparent continuity of grain morphology observed in LM images is consistent with this interpretation; however, quantitative EBSD orientation and misorientation analyses would be required to confirm epitaxial growth (Figure 10).

The observed thin reaction layer at the interface, characterized by elemental segregation, fine phases, and micro-regions of solid solutions, forms in Step 5 as a result of ongoing diffusion and segregation processes.

Final step: The subsequent cooling of the structure leads to solidification, which in LM observations manifests as grain boundaries appearing to continue across the interface from the copper side.

The EBSD observations suggest that the crystallographic characteristics of the brazed joint may be influenced by the adjacent substrate. One possible explanation is substrate-assisted nucleation followed by crystal growth into the filler metal. Such a mechanism could involve an epitaxial contribution, provided that a suitable crystallographic relationship exists between the substrate and the growing crystals. However, the present EBSD results do not provide sufficient quantitative evidence to confirm epitaxial growth, as crystallographic misorientation and orientation relationships across the interface have not been systematically established. Therefore, substrate-assisted nucleation and possible epitaxial growth are considered plausible mechanisms that require further investigation.

## 5. Integrated Mechanism of Filler Metal Formation in the Cross-Joint

**Figure 11:**
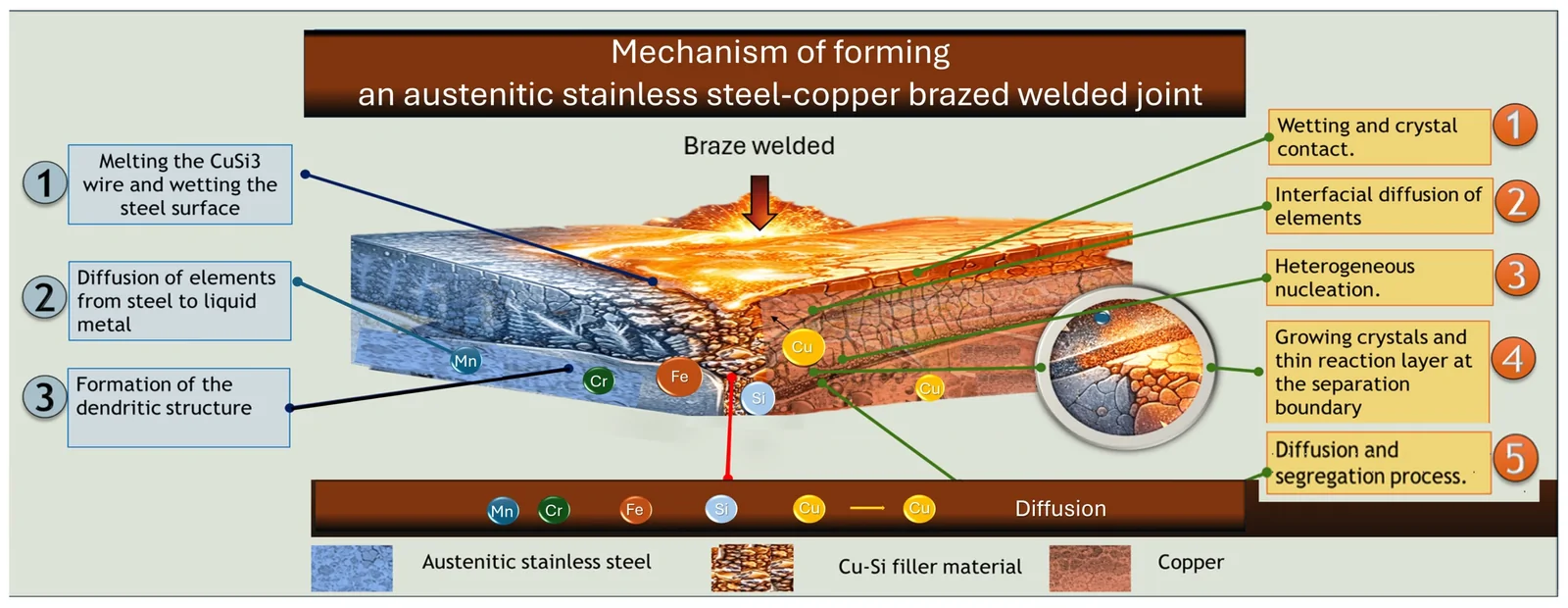
Proposed mechanism of forming braze-welding joint (own research).

The proposed stages of cross-joint formation between austenitic steel and copper, obtained by brazing using CuSi3 filler wire, are presented in Figure 11.

The proposed mechanism is consistent with information dispersed throughout the literature (a detailed analysis is presented in the preceding chapters). In many brazed systems, the molten metal penetrates the grain boundaries of the base material, which can result in a visual mapping of the structure. Factors contributing to the observed phenomenon include the following:

- Small differences in the lattice parameters of the FCC structure of the austenitic steel under study and copper;
- High wettability of the substrate materials;
- The adopted parameters of the braze-welding process (process thermodynamics), ensuring that the residence time at sufficiently high temperature is adequate for intensive grain boundary diffusion to occur.

When analyzing the proposed mechanism (Figure 5) of braze joint formation, attention must be paid to the complexity of the diffusion processes occurring during joint development. Diffusion processes occur at different stages of structure formation during brazing (stage 2 from the steel side and stages 2–5 from the copper side). Initially, after supplying the system with sufficient thermal energy (beginning of brazing, stage 1—melting), the potential barrier surrounding the atoms is overcome. This energy represents the activation energy for diffusion. With increasing temperature, atomic mobility generally increases, facilitating diffusion through vacancy-mediated and, where applicable, interstitial mechanisms. Although interstitial diffusion of elements such as carbon and oxygen is theoretically possible, these processes were not directly investigated in the present study and are considered only as potential mechanisms based on literature data.

Thus, even after slight cooling of the joint, additional diffusion mechanisms appear. During braze formation (stages 2–5 in the schematic), specific mechanisms operate related to the volume of lattice defects represented by grain boundaries (stages 2–4), as well as the formation of compounds involving reactive diffusion (stage 5) and the development of solid solutions. Grain boundaries are surface defects, and the atomic packing density in these regions is lower than in the lattice, facilitating atom jumps. The role of grain boundaries as fast diffusion pathways depends on the boundary type. The contribution of grain boundaries to diffusion depends on their crystallographic character, structure, temperature, and the diffusing species. According to the Arrhenius relationship, a lower activation energy generally corresponds to a higher diffusion coefficient at a given temperature, assuming comparable pre-exponential factors. High-angle grain boundaries may provide preferential diffusion pathways; however, their relative contribution cannot be established without quantitative grain-boundary characterization. Therefore, grain-boundary diffusion is considered a possible mechanism based on literature data rather than a process directly confirmed in the present investigation.

Stage 5 involves possible interfacial reactions and further elemental redistribution. The SEM/EDS observations indicate local compositional variations, which may be associated with the development of solid solutions or other reaction products. However, the formation of specific intermetallic compounds was not conclusively confirmed by the available crystallographic data. Therefore, their possible formation remains a literature-based hypothesis rather than an experimentally verified result. The final stage involves completion of solidification and development of the observed microstructure. The presented results indicate that the governing factors in the proposed mechanism of braze-weld formation are the process temperature and brazing speed [63] (which determines the high-temperature diffusion time), as well as the crystal lattice type of the joined materials. To a lesser extent, the driving force of chemical diffusion (diffusion according to Fick’s first and second laws) in this system is the concentration gradient- the difference in elemental concentrations. This is because the chemical composition of copper and electrode wires is very similar, significantly limiting this type of diffusion, whose rate diminishes over time as the concentration gradient equilibrates. Moreover, copper does not dissolve carbon. It should also be noted that Fick’s equations fundamentally apply to binary systems rather than multicomponent systems. When a third (or further) component is present, a modified form of Fick’s equations applies, in which chemical potential gradients replace concentration gradients. Literature analysis [63,64,65,66] confirms that the third component can influence the chemical potentials of the remaining two components in a differentiated manner; consequently, even in the absence of a concentration gradient of a given component, a chemical potential gradient may exist, driving chemical diffusion. Additionally, it should be noted that the mutual influence of elements on chemical potentials is enhanced by a temperature gradient. However, changes in chemical potentials usually require prolonged exposure to high or elevated temperatures, as in long-term annealing. This fact significantly limits (although does not entirely preclude) the possibility of chemical diffusion during the braze-welding process under study.

## 6. Conclusions

The experimental results obtained for MIG braze-welded Cu–AISI 304 stainless-steel joints lead to the following conclusions:

1. Joint quality was strongly affected by the specific combination of welding current, arc voltage, and travel speed within the investigated process window of 150–170 A, 19–21 V, and 220–320 mm/min. Defect-free joints were obtained for samples n3, n4, and n8, whereas other parameter combinations resulted in insufficient wetting, porosity, or interfacial cracking.
2. Linear heat input alone was not sufficient to uniquely predict joint quality. For example, defect-free and defective joints were obtained at relatively similar calculated heat-input values, indicating that the manner in which energy was delivered through the particular combination of current, voltage, and travel speed also influenced joint formation. This conclusion is limited to the investigated parameter set and should not be interpreted as a statistically validated universal process relationship.
3. The mean ultimate tensile strength of the joints meeting the adopted NDT acceptance criterion ranged from 123.2 to 130.8 MPa. The highest mean value (130.8 ± 0.84 MPa) was obtained for configuration n4, produced at 170 A, 19 V, and 320 mm/min. One-way ANOVA confirmed statistically significant differences among the three investigated configurations (F(2, 12) = 127.52, *p* < 0.001). Post hoc comparisons demonstrated that configuration n4 exhibited significantly higher tensile strength than both n3 and n8, whereas no statistically significant difference was observed between n3 and n8. These findings identify n4 as the most favorable configuration in terms of tensile strength among the three investigated conditions.
4. On the stainless-steel side, a sharp interface with a thin Fe–Cu–Si-enriched region and dendritic Cu-based filler-metal structure was observed. On the copper side, microstructural observations revealed an apparent continuity of grain morphology across the interface, suggesting differences in local solidification and interfacial growth behavior. However, further quantitative crystallographic analysis is required to confirm the proposed growth mechanisms.
5. Based on the combined LM, SEM/EDS, and EBSD observations, a qualitative joint-formation mechanism involving wetting, interfacial diffusion, solidification, and side-dependent microstructural evolution is proposed. Because no temperature–time history or diffusion coefficients were measured, the proposed diffusion sequence should be regarded as a metallurgical interpretation rather than a quantitatively validated kinetic model.

The present study was limited to a relatively narrow process window with two levels of current, voltage, and travel speed. Quantitative diffusion coefficients and temperature–time cycles were not measured, and additional phase-identification methods would be required to unambiguously confirm the nature and fraction of interfacial phases. Further work should also include replicated statistical analysis of microstructural dimensions and mechanical properties over a broader range of processing conditions. The present results therefore describe joint formation and mechanical behavior under the investigated laboratory conditions and should not be interpreted as evidence of corrosion resistance, fatigue performance, leak-tightness, residual-stress state, or long-term marine-service durability.

## Figures and Tables

**Table 1:** Main bonding mechanisms presented in the literature.

Mechanism	Material System	Primary Physical Phenomenon	Cu Role	Austenitic Steel Role	Model Limitation	Source (DOI)
Liquid diffusion + IMC	Cu/steel + Sn-based solder	Cu diffusion → solder + IMC formation	Active atom donor	Pasive diffusive barrier	Neglect of Interface Asymmetry	[17] (Szczucka-Lasota et al., 2025)
Solid-state diffusion	Cu/Ni/steel	Mutual diffusion + transition zones	Rapid diffusion	Slower diffusion (Fe, Cr)	Lack of Multicomponent Kinetic Model	[18] (Sebastian & Suyamburajan, 2020)
Diffusion without IMC (Cu interlayer)	Cu/steel 304	Solid solution + lattice distortion	Bonding Mediator	Strengthening via deformation	Lack of nucleation description	[18] (Sebastian & Suyamburajan, 2020)
SLID (solid–liquid interdiffusion)	Cu/Cu (reference model)	Phase reactions + IMC growth	Predominant component	none	No reference to steel	[19] (Liu et al., 2024)
Active brazing	steel/Ag-Cu alloy	multilayer IMC	Formation of Cu-Ti phases	Fe-Ni reactions	Lack of diffusion–crystallization coupling	[20] (Laik et al., 2013)
Grain boundary diffusion	metal-metal systems	IMC ∝ t0.5 growth	Rapid migration	Limited diffusion	Lack of 3D model	[21] (Zhu et al., 2024)
SSW review model	Steel—various types	Diffusion mechanisms	Temperature dependent	Structure—controlled	No copper-specific consideration	[22] (Khedr et al., 2023)
Ag-Cu-Pd brazing	316L steel	Wettability improvement	Diffusion improvement	IMC limitation	Lack of kinetic model	[23] (Wang et al., 2023)

**Table 2:** Mechanical properties of base materials.

Material	UTS, MPa	HV
(CW004A), 99.9% Cu, (EN1652:1997)	205	75
Austenitic steel 304 (UNS S30400)	615	150

**Table 3:** Braze welded materials—physical properties.

Material	Electrical Conductivity, σ [MS/m]	Thermal Conductivity λ [W/(m·K)]
(CW004A), 99.9% Cu	58	390
Austenitic steel 304	1.39	16.2

**Table 4:** The selected properties of electrode wires.

Electrode Wire	Main Elements	Melting Point, °C
CuSi3		
(EN ISO 24373: S Cu 6560 (C)/AWS ERCuSi-C)	Cu + 3% Si + 1% Mn	1025
CuSn6 EN ISO 24373; S Cu 5180/AWS A5.7 → ERCuSn-A	Cu + 6% Sn	1005

**Table 5:** NDT parameters.

Testing Method	Main Equipment Information	Testing Parameters
VT	Torch, magnifying glass, weld gauge, lux meter, caliper	Illumination: 750 lx
PT	Penetrans: BDS, BRE-S, BEA	Penetration time: 30 min, Development time: 30 min, Illumination: 750 lx,

**Table 6:** Sample designation for braze welding samples using CuSi3 electrode wire.

Sample (Name of Group)	Current, A	Voltage, V	Welding Speed, mm/min	NDT Results	Sample	Current, A	Voltage, V	Welding Speed, mm/min	NDT Results
n1	150	19	220	The binder does not spread evenly across the stainless-steel surface.	n1-1	−1	−1	−1	0
					n1-2	−1	−1	−1	0
					n1-3	−1	−1	−1	0
					n1-4	−1	−1	−1	0
					n1-5	−1	−1	−1	0
n2	150	19	320	Lack of proper wetting.	n2-1	−1	−1	+1	0
					n2-2	−1	−1	+1	0
					n2-3	−1	−1	+1	0
					n2-4	−1	−1	+1	0
					n2-5	−1	−1	+1	0
n3	170	19	220	Correct joint	n3-1	+1	−1	−1	1
					n3-2	+1	−1	−1	1
					n3-3	+1	−1	−1	1
					n3-4	+1	−1	−1	1
					n3-5	+1	−1	−1	1
n4	170	19	320	Correct joint	n4-1	+1	−1	+1	1
					n4-2	+1	−1	+1	1
					n4-3	+1	−1	+1	1
					n4-4	+1	−1	+1	1
					n4-5	+1	−1	+1	1
n5	150	21	220	Blisters and porosity.	n5-1	−1	+1	−1	0
					n5-2	−1	+1	−1	1
					n5-3	−1	+1	−1	0
					n5-4	−1	+1	−1	0
					n5-5	−1	+1	−1	0
n6	150	21	320	Interfacial cracks. Cracks at the copper-binder interface.	n6-1	−1	+1	+1	0
					n6-2	−1	+1	+1	0
					n6-3	−1	+1	+1	0
					n6-4	−1	+1	+1	0
					n6-5	−1	+1	+1	0
n7	170	21	220	Gas bubbles in the weld zone or near the braze joint boundary.	n7-1	+1	+1	−1	0
					n7-2	+1	+1	−1	0
					n7-3	+1	+1	−1	0
					n7-4	+1	+1	−1	0
					n7-5	+1	+1	−1	0
n8	170	21	320	Correct joint	n8-1	+1	+1	+1	1
					n8-2	+1	+1	+1	1
					n8-3	+1	+1	+1	1
					n8-4	+1	+1	+1	1
					n8-5	+1	+1	+1	1

**Table 7:** Calculation of Effective Linear Energy [J/mm].

Sample	$Q_{\mathrm{electric}}=\frac{I\cdot U\cdot 60}{V}$ [**J**/**mm**]	***Q_real_*** = 0.8·***Q_electric_*** [**J**/**mm**]	Measurement Error ΔQ [J/mm]	Relative Error [%]	Standard Deviation
n1	777.27	621.82	7.94	1.02	7.3
n2	534.38	427.50	4.65	0.87	3.3
n3	880.91	704.73	7.75	0.88	6.6
n4	605.63	484.50	4.92	0.81	3.6
n5	859.09	687.272	7.51	0.87	10.6
n6	590.63	472.504	4.41	0.75	6.2
n7	973.64	778.912	7.31	0.75	10.3
n8	669.38	535.50	4.67	0.70	6.6

**Table 8:** factor-p correlation table.

Effect	β Factor	*p*
Current (A)	+1.636	0.0024
Voltage (B)	−0.437	0.3864
Speed (C)	+0.437	0.3864
A × B	−0.762	0.1287
A × C	+0.762	0.1287
B × C	+0.437	0.3864
A × B × C	+0.762	0.1287

**Table 9:** Metallurgical mechanisms responsible for the samples group rejection.

Metallurgical Mechanism	Cause	Sample
Porosity resulting from trapped gases	Insufficient time for gas release; process conducted at excessive speed	n5;n7
Interfacial cracks resulting from differences in the thermal expansion coefficients of Cu and the filler metal	Thermal stresses during cooling, initially caused by excessively high temperature (too high current–voltage parameter values)	n6
Lack of wettability and high interfacial energy	Temperature too low, not exceeding the critical value for wetting	n2

**Table 10:** Tensile strength results UTS versus linear power velocity.

Joint Symbol	Parameters of Braze-Welding	Linear Power Velocity	Results UTS, MPa Measurement:					UTS, MPa (Average Value)	Standard Deviation	CV
			1	2	3	4	5			
n3	170 A/19 V/220 mm/min	14.68 W mm/min	124	123	122	124	123	123	0.84	0.679%
n4	170 A/19 V/320 mm/min	10.09 W mm/min	131	130	131	132	130	131	0.84	0.640%
n8	170 A/21 V/320 mm/min	11.16 W mm/min	125	123	123	124	124	124	0.84	0.676%

**Table 11:** ANOVA pair test results.

Comparison	Mean Difference	95% Simultaneous CI (Tukey)	Holm-Adjusted *p*
n4 − n3	+7.60 MPa	6.19 to 9.01	1.91 × 10^−8^
n4 − n8	+7.00 MPa	5.59 to 8.41	3.24 × 10^−8^
n8 − n3	+0.60 MPa	−0.81 to 2.01	0.279

## Data Availability

The original contributions presented in this study are included in the article. Further inquiries can be directed to the corresponding authors.
